# Small UAS-Based Wind Feature Identification System Part 1: Integration and Validation

**DOI:** 10.3390/s17010008

**Published:** 2016-12-23

**Authors:** Leopoldo Rodriguez Salazar, Jose A. Cobano, Anibal Ollero

**Affiliations:** Robotics, Vision and Control Group, Universidad de Sevilla, 41092 Sevilla, Spain; jcobano@us.es (J.A.C.); aollero@us.es (A.O.)

**Keywords:** wind prediction, wind estimation, UAS, wind shear, gust, multi-platform integration

## Abstract

This paper presents a system for identification of wind features, such as gusts and wind shear. These are of particular interest in the context of energy-efficient navigation of Small Unmanned Aerial Systems (UAS). The proposed system generates real-time wind vector estimates and a novel algorithm to generate wind field predictions. Estimations are based on the integration of an off-the-shelf navigation system and airspeed readings in a so-called direct approach. Wind predictions use atmospheric models to characterize the wind field with different statistical analyses. During the prediction stage, the system is able to incorporate, in a big-data approach, wind measurements from previous flights in order to enhance the approximations. Wind estimates are classified and fitted into a Weibull probability density function. A Genetic Algorithm (GA) is utilized to determine the shaping and scale parameters of the distribution, which are employed to determine the most probable wind speed at a certain position. The system uses this information to characterize a wind shear or a discrete gust and also utilizes a Gaussian Process regression to characterize continuous gusts. The knowledge of the wind features is crucial for computing energy-efficient trajectories with low cost and payload. Therefore, the system provides a solution that does not require any additional sensors. The system architecture presents a modular decentralized approach, in which the main parts of the system are separated in modules and the exchange of information is managed by a communication handler to enhance upgradeability and maintainability. Validation is done providing preliminary results of both simulations and Software-In-The-Loop testing. Telemetry data collected from real flights, performed in the Seville Metropolitan Area in Andalusia (Spain), was used for testing. Results show that wind estimation and predictions can be calculated at 1 Hz and a wind map can be updated at 0.4 Hz. Predictions show a convergence time with a 95% confidence interval of approximately 30 s.

## 1. Introduction

Current UAS technology has advanced in such a way that any unexperienced user is able to plan a route with relatively good accuracy. As the reliability has increased, applications using small UAS are growing rapidly. In addition, nonlinear natural effects, such as winds, can be compensated even with Commercial-Off-The-Shelf (COTS) components. Nevertheless, to compensate wind effects efficiently, the use of a sensor that can provide wind measurements is sometimes limited by the platform payload and the cost. This leads to inefficient attitude compensations, producing drift and sometimes missing waypoints, which may result likely into higher energy consumptions [1]. Currently, there are several research efforts to provide wind estimations without a direct measurement of the wind. Langelaan, et al. [2] proposed two ways of estimating the wind field, both using measurements from a standard sensor suite, i.e., Inertial Measurement Unit (IMU) and Global Navigation Satellite System (GNSS). The first method consists in a comparison between predictions generated with a dynamic model and actual measurements of the aircraft motion. The second one consists in the estimation on wind acceleration and its derivatives from the GNSS velocity, i.e., using the pseudorange rate of change together with direct measurements of the vehicle acceleration. Johansen, et al. [3] have developed a method in which the wind is estimated using an observer which leads to the calculation of sideslip and Angle-of-Attack (AOA). They estimate the wind from the difference between the platform velocity relative to the wind and the velocities in the body frame utilizing a Kalman Filter, which is a similar approach than the one presented in [4]. Other approaches, such as the one presented by Larrabee et al. [5] uses flow angle sensors and a Pitot tube with two Unscented Kalman Filters (UKF). This innovation compares information from different platforms in order to produce real time estimates. Neummann & Bartholmai [6] produced wind estimations with a quadcopter UAS without the use of any additional sensors rather than its standard sensor suite, even without a dedicated airspeed sensor, and/or anemometer, based mainly in the wind triangle and the vector difference between the ground speed and an estimated speed. Condomines, et al. [7] have published a set of results of a flight campaign with estimations of the wind field considering non-linear wind estimation with an square-root UKF in which the platform was equipped with an standard sensor suite which provides measurements that estimate angle of attack and sideslip.

Previously, as part of this research effort, the authors have introduced an algorithm that can estimate the wind field in such way that the different wind features (gust, shear, etc.) can be identified separately with a method that calculates statistical properties and based on distribution models of the wind, such as the 1-cos model for gusts and the wind shear model [8,9]. Lawrance & Sukkarieh [4] propose a method that incorporates a Gaussian regression in order to predict within a limited amount of time (up to 10 s) the local wind field despite the feature that is present. The identification of features, such as shear, thermals [10], gusts is of particular importance in the so-called atmospheric energy harvesting [4]. On this field, several authors such as Cutler et al. [11] and Chakrabarty et al. [12] have published successful results on the generation of static soaring trajectories and others, such as Montella & Spletzer [13] and Bird et al. [14] have developed systems that produce and follow dynamic soaring trajectories. In addition, Bencatel et al. [15] have performed an analysis on necessary conditions for dynamic soaring and how this problem can be seen as a function of aircraft and environmental parameters. Despite the advances in the generation of the trajectories (Rucco et al. [16]), there are few methods for identifying wind features, and the creation of real-time algorithms for energy harvesting should be addressed and improved. A few authors have described the integration of such methods in a level of detail that can identify areas of opportunities in hardware selection, software architecture, computational time, etc.

This paper presents and describes the detailed integration at a hardware and software level of the system. This enables the estimation of the wind vector and identification of the features in real-time with a standard sensor suite. In the previous works of the authors [8,9], the identification system was firstly introduced. In the work presented, wind features (wind shear and discrete gusts) are identified separately based on statistical analysis by fitting wind estimates into a Weibull distribution. The wind identification system allows the generation of a 3-dimensional wind map with predictions of what the wind vector would be at a certain location. The system presents innovations regarding its architecture, and adds the capability for continuous gust identification. Preliminary results are presented in two stages: simulations of the different features and a Software-In-The-Loop (SITL) testbed fed up with previous flight information. The verification with actual experiments is going to be presented in a follow-up manuscript.

The paper is organized as follows: Section 2 presents a brief summary of the methods and statistical analysis utilized. Section 3 describes the hardware and software architecture of the system. Section 4 shows the validation results of the different components. Section 5 presents a discussion on the obtained results. Finally, conclusions are presented in Section 6.

## 2. Wind Field Estimation and Wind Field Prediction

The generation of the wind field considers both the estimation and prediction processes. Both are equally important, however they not necessary have to occur at the same time and rate. This section provides the insights of the selected methods for these operations.

### 2.1. Wind Field Estimation

The selected method for estimation process was originally presented in [2]. It estimates the wind without the use of an observer (Kalman or Particle filters) by using the velocity vector calculated by the GNSS module together with measurements of the vehicle acceleration and a portion of the state vector of the platform. The goal is to calculate the wind acceleration and velocity using the relationship between the GNSS velocity and the body-axis state from the COTS Autopilot Module (APM).

**Figure 1:**
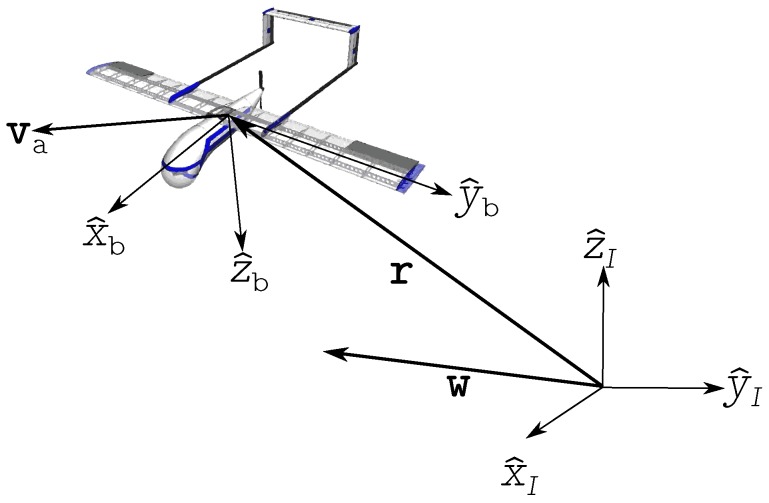
Reference frames utilized in the formulation of the identification of wind vector problem.

Consider a UAS located in r in the inertial frame I. The unit vectors of this frame are defined as $\left({\hat{x}}_{I},{\hat{y}}_{I},{\hat{z}}_{I}\right)$. Consider also a body frame b with unit vectors $\left({\hat{x}}_{b},{\hat{y}}_{b},{\hat{z}}_{b}\right)$ with its origin at the center of mass of the vehicle. The wind vector w and the airmass-relative velocity $v_{a}$ are illustrated in Figure 1.

The velocity of the vehicle expressed in the inertial frame is.

(1) $$\dot{r}=v_{a}+w$$

From Equation (1), two relationships are used to characterize the instantaneous wind vector. Further details on the derivation of these relationships can be found in [2,8,9].

The first one indicates the correspondence between the vehicle kinematics and the GNSS velocity expressed with respect to the **I** frame:

(2) $${\left[\begin{array}{c} w_{x}\\ w_{y}\\ w_{z} \end{array}\right]}_{I}={\left[\begin{array}{c} \dot{x}\\ \dot{y}\\ \dot{z} \end{array}\right]}_{I_{\mathrm{GNSS}}}-{\left(C_{I}^{b}\right)}^{-1}{\left[\begin{array}{c} u\\ v\\ w \end{array}\right]}_{b}$$

where ${\left(w_{x},w_{y},w_{z}\right)}_{I}$ is the wind velocity vector, ${\left(\dot{x},\dot{y},\dot{z}\right)}_{I_{\mathrm{GNSS}}}$ is the GNSS velocity vector and ${\left(u,v,w\right)}_{b}$ are the components of the velocity with respect the air mass expressed in the body frame and assumed to be calculated by the autopilot. $C_{I}^{b}$ is the Direction Cosine Matrix, which transforms a vector expressed in the inertial frame to one expressed in the body frame.

The second relationship aims to calculate the wind acceleration expressed in the body frame at the previous step (k-1). Since the IMU body-axis accelerations expressed in the body frame are given by.

(3) $${\left[\begin{array}{c} a_{x}\\ a_{y}\\ a_{z} \end{array}\right]}_{b}={\left[\begin{array}{c} {\dot{w}}_{x}\\ {\dot{w}}_{y}\\ {\dot{w}}_{z} \end{array}\right]}_{b}+{\left[\begin{array}{c} \dot{u}\\ \dot{v}\\ \dot{w} \end{array}\right]}_{b}+\left[\begin{array}{c} qw-rv+g\sin\theta \\ ru-pw-g\cos\theta \sin\phi \\ pv-qu-g\cos\theta \cos\phi \end{array}\right]+b_{\mathrm{imu}}+n_{\mathrm{imu}}$$

where $\left(a_{x},a_{y},a_{z}\right)$ is the body axis accelerations vector, (p,q,r) is the rotation rate vector, *g* is the gravity force, *θ* is the roll angle, *ϕ* is the pitch angle, $b_{\mathrm{imu}}$ is the accelerometer bias and $n_{\mathrm{imu}}$ is the white noise from the IMU.

Since the calculation of the rate of change of the velocity with respect the air mass can’t be determined with the on-board sensors it can be estimated with a second order numerical differentiation. Therefore, the wind speed rate of change at the previous step k-1 was derived:

(4) $${\left[\begin{array}{c} {\dot{w}}_{x}\\ {\dot{w}}_{y}\\ {\dot{w}}_{z} \end{array}\right]}_{b,k-1}={\left[\begin{array}{c} a_{x}\\ a_{y}\\ a_{z} \end{array}\right]}_{b,k-1}-{\left[\begin{array}{c} b_{x}\\ b_{y}\\ b_{z} \end{array}\right]}_{k-1}+{\left[\begin{array}{c} -g\sin\theta \\ g\cos\theta \sin\phi \\ g\cos\theta \cos\phi \end{array}\right]}_{k-1}-{\left[\begin{array}{c} qw-rv\\ pw-ru\\ qu-pv \end{array}\right]}_{k-1}-\frac{1}{2\Delta t}\left[\begin{array}{c} u_{k}-u_{k-2}\\ v_{k}-v_{k-2}\\ w_{k}-w_{k-2} \end{array}\right]$$

Equations (2) and (4) are necessary for trajectory planning. Using different sources of error increases the reliability of the solution. Moreover, the calculation of wind acceleration and velocity based on actual inertial and GNSS measurements ensures bounded errors which is a key advantage compared to other methods (e.g., the use of a dynamic model).

### 2.2. Wind Field Prediction

The wind field could be estimated at each time step from the data provided by the IMU, GNSS and the vehicle dynamics as shown in Section 2.1. Previous results [8,9] show that the estimation algorithm produce accurate results. However, these estimations are not sufficient if the information is going to be used for precise trajectory planning. Therefore, a prediction stage is needed so that the wind field could be inferred within a reasonable time window.

In this context, three models of different wind features have been selected: the wind shear model, the discrete gust model and the continuous (Dryden) wind turbulence model. These are widely used in the aerospace industry and are contained in the Military Specification MIL-F-8785C [17] and Military Handbook MIL-HDBK-1797 [18].

#### 2.2.1. Wind Shear Model

The magnitude of the wind is modeled by the following equation:

(5) $$W_{\mathrm{shear}}=W_{20}\frac{\ln\frac{h}{z_{0}}}{\ln\frac{6.096}{z_{0}}},1\;m<h<300\;m$$

where $W_{\mathrm{shear}}$ is the mean wind speed, $W_{20}$ is the wind speed at 20 ft (6.096 m) and $z_{0}$ varies depending on the flight phase. However, a value of 0.0457 m (0.15 ft) is selected due to the characteristics of the platform, i.e., flight below 1000 ft. Finally, *h* is the actual altitude of the vehicle.

**Figure 2:**
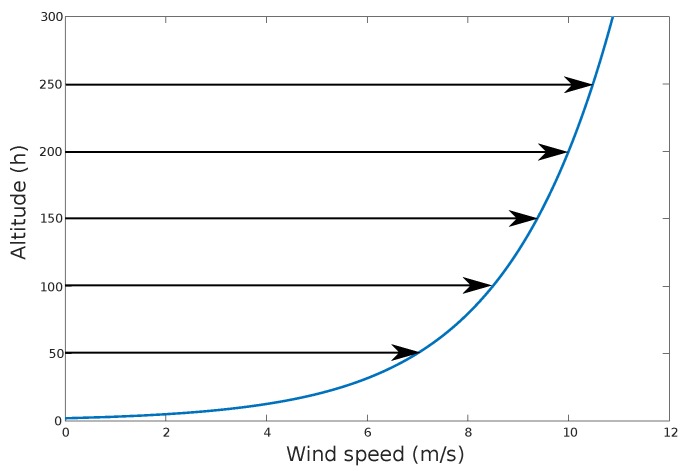
Typical shear profile that shows the increase of wind speed over as the altitude increases. The relationship is exponential between the two variables.

The wind shear is illustrated in Figure 2.

In order to characterize the wind shear, it is assumed that the wind varies with the altitude following the Prandtl Ratio based on an Empirical Power Law (EPL) [8]:

(6) $$\frac{W_{1}}{W_{2}}={\left(\frac{h_{1}}{h_{2}}\right)}^{\xi }$$

where *ξ* is the Prandtl coefficient that shapes the EPL function. $\left(W_{1},W_{2}\right)$ are two wind speeds and $\left(h_{1},h_{2}\right)$ are the corresponding altitudes.

#### 2.2.2. Discrete Gust Model

This model uses the implementation of the 1-cos shape and its mathematical representation is as follows:

(7) $$W_{\mathrm{gust}}=\left\{\begin{array}{cc} 0 & x<0\\ \frac{W_{m}}{2}\left(1-\cos\frac{\pi x}{d_{m}}\right) & 0\leq x\leq d_{m}\\ W_{m} & x>d_{m} \end{array}\right.$$

where $W_{m}$ is the magnitude of the gust and $d_{m}$ is the gust length and *x* is the distance traveled.

**Figure 3:**
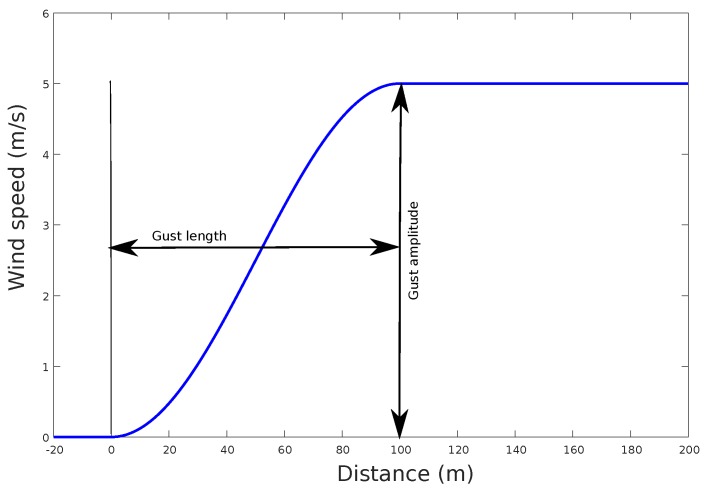
Typical discrete gust profile that shows a growth over the wind on a short period of time from the initial wind speed of the gust magnitude, and a permanent increase at the end of the gust length.

The discrete gust is illustrated in Figure 3.

#### 2.2.3. Continuous Gust Model

The selected model for continuous gust utilizes the Dryden spectral representation in which the turbulence is considered a stochastic process defined by velocity spectra. In [17,18,19], the power spectral densities are defined. Note that for simulation purposes the Low-Altitude scale lengths have been used.

The number of variables in the continuous gust model is vast. Therefore, inferring these values from actual wind measurements trough a regression is very complex. Thus, this model is used only as a simulation input.

Two methods are proposed for continuous gust identification in both short and long term. The first one incorporates a Standard Gaussian Process (GP) Regression [20].

Considering a set of vertical wind observations of size $\bar{M}$, ${\hat{W}}_{z}={\hat{W}}_{z,i}{|}_{i=1}^{\hat{M}}$. The wind speed prediction $\bar{W_{p}}\left(x\right)$ at any location *x* can be expressed as:

(8) $$\bar{W_{p}}\left(x\right)=\sum_{i=1}^{\bar{M}}k_{i}{\hat{W}}_{z,i}$$

in which $k_{i}$ is the *i*-th coefficient of the linear combination of wind measurements ${\hat{W}}_{z}$. Based on the work presented by Park et al. [21], an optimal coefficient is determined by minimizing the prediction error.

(9) $$\min_{k}E\left[{\left(\bar{W_{p}}\left(x\right)-W_{p}\left(x\right)\right)}^{2}\right]=\min_{k}\left(k^{T}\left[Q\left(X,X\right)+\sigma_{n}^{2}I\right]k-2k^{T}q\left(X,x\right)+q\left(x,x\right)\right)$$

which can be determined by calculating the covariance matrix Q(X,X) and the covariance vector q(X,x) between every two observations at locations X and *x*; finally q(x,x) represents the covariance value . This leads to express standard GP regression of the linear predictor as:

(10) $$\bar{p}\left(X\right)=k^{T}{\hat{W}}_{z}=q\left(x,X\right){\left[Q\left(X,X\right)+\sigma_{n}^{2}I\right]}^{-1}{\hat{W}}_{z}$$

and the covariance value $cov(\bar{p}\left(x\right)))$ can be expressed as:

(11) $$cov\left(\bar{p}\left(x\right)\right)=q\left(x,x\right)-q\left(x,X\right){\left[Q\left(X,X\right)+\sigma_{n}^{2}I\right]}^{-1}q\left(x,X\right)$$

where $\sigma_{n}^{2}$ is the measurement noise covariance.

An alternative approach can be used to perform long-term predictions by employing a non homogeneous regression prediction model. Lerch and Thoraninsdottir [22] have performed a comparison between three non-homogeneous regression model, which allows to produce predictions in day time window. Since the intention of the intended testing flight campaigns is to store data into a single database, a big amount of data can be utilized to perform the predictions with the selected regression model.

At this stage, the truncated normal model was selected as a form of wind estimation. Being *W* the wind speed and $X_{1},\cdots ,X_{j}$ the ensemble member forecasts, the predicted distribution of *W* can be approximated by a truncated normal distribution:

(12) $$W|X_{1},\cdots ,X_{j}∼N_{\left[0,\infty \right)}\left(\mu ,\sigma^{2}\right)$$

where the mean *μ* is an affine function of the ensemble forecast and the variance $\sigma^{2}$ is an affine function of the ensemble variance. If these exchange members are exchangeable [22], the distribution function of the Truncated Normal (TN) distribution F(z) is given by:

(13) $$F\left(z\right)=\Phi {\left(\frac{\mu }{\sigma }\right)}^{-1}\Phi \left(\frac{z-\mu }{\sigma }\right)$$

for z>0, where Φ is the cumulative standard normal distribution.

This is indeed a simple non-homogeneous method. However, results indicate that the training period to produce accurate predictions in one-day ahead forecasts is of the equivalent 30 days of continuous measurements [22].

#### 2.2.4. Weibull Distribution

The Weibull distribution is a key part of the research performed as many datasets, including wind speed have been proved to fit in. The Weibull distribution has three main parameters, the shaping factor *κ*, the scaling factor *ν* and the threshold. Given a dataset **W** $=(W_{1}...W_{n})$, the Weibull probability density function can be expressed as a function of a wind magnitude *W* [8]:

(14) $$f\left(W\right)=\frac{\kappa }{\nu }{\frac{W}{\nu }}^{\kappa -1}e^{\frac{W}{\nu }\kappa }$$

From this function the most probable wind speed $W_{\mathrm{mp}}$ at a particular location can be expressed in terms of the Weibull parameters:

(15) $$W_{\mathrm{mp}}=\nu {\left(1-\frac{1}{\kappa }\right)}^{\frac{1}{\kappa }}$$

**Figure 4:**
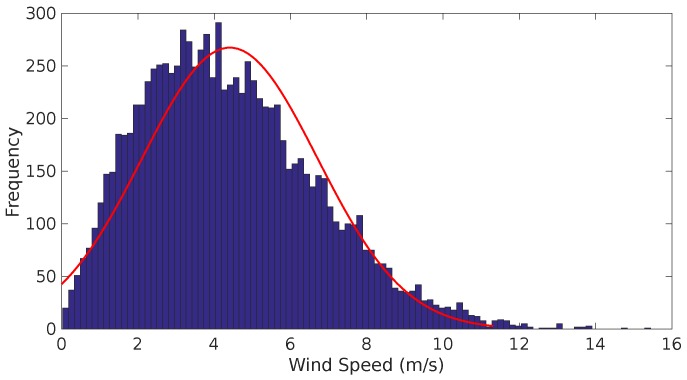
Typical weibull distribution from a National Oceanic and Atmospheric Administration (NOAA) measurement station at an altitude of 30 m.

A typical Weibull distribution is shown in Figure 4.

#### 2.2.5. Genetic Algorithm and the Weibull Distribution Parameters

Genetic Algorithm is a searching method that simulates the evolution theory. The method aims to generate possible random solutions (chromosomes) to a problem stated in a for of an objective function (fitness function). A given set of chromosomes is a population in a generation. Every one of them will produce evolved chromosomes based on three operations: reproduction, crossover and mutation. Details in the implementation of the GA can be found in [23].

In order to calculate the shaping parameter *κ* of a Weibull-distributed data set, one has to calculate the residual error *ϵ* between the measured mean and the standard deviation of the wind estimates (see Section 2.1) and a theoretical mean and standard deviation derived from the Weibull distribution moment, as stated in the following equation:

(16) $$\epsilon =\sigma^{2}/\mu^{2}-\frac{\Gamma \left(1+2/\kappa \right)+\Gamma^{2}\left(1+1/\kappa \right)}{\Gamma^{2}\left(1+1/\kappa \right)}$$

where Γ is the gamma function *σ* is the standard deviation of the wind estimates and *μ* is the mean of the wind estimates.

Once Equation (16) converges to a desired tolerance value, an acceptable *κ* value is obtained and the scaling parameter can be calculated based on the following equation:

(17) μ=υΓ(1+1/k)

### 2.3. Wind Mapping

In order to generate a full 3D wind map, a combination of methods is required. Initially, the work presented in [8] suggests the use of a Newton polynomial extrapolation in order to generate local values of the Prandtl coefficient, *ξ*, from which the shaping and scaling parameters can be calculated in order to obtain a local most probable wind speed $W_{\mathrm{mp}}$. This is true in case of the presence of a wind shear. However, if a gusts is detected, the extrapolation will accumulate error, producing an inaccurate wind map.

Therefore, a 3D map can be generated based on the feature that is detected. The GP regression shown in Section 2.2.3 allows the generation of a wind map with predictions based on the estimates found at position *X*. These estimates carry information of the covariance which is continuously updated with the different feature detection algorithms. Details on the wind mapping algorithm and the results are to be found in the Part 2 of this research.

## 3. System Architecture

This section describes the hardware, software and communication architecture of the wind identification system.

The selected hardware takes mainly two COTS components in order to perform the estimation and the prediction of the wind field. The selected autopilot is the Pixhawk (3D Robotics, Berkeley, CA, USA) which is based on the PX4 open-hardware project. The characteristics of this module can be found in [24]. Given the processor characteristics, the wind estimation and wind prediction algorithms have to reside in a dedicated computer. The selected computer is the ODROID-C2 (Hardkernel, Anyang Gyeonggi-do, Korea) [25] that contains a quad-core processor at 2 Ghz at 64 bit. The main characteristics are enumerated in Table 1.

The required algorithms need an additional platform that shall do the data analysis of the stored variables. All the wind estimates and predictions are kept in a database. As more flights are to be performed as part of the validation, verification and other applications, the wind database will grow. Due to its size and for reliability, a ground station contains the wind prediction and estimation database. A PC with an ©Intel Core(TM) i755000U CPU (Seattle, WA, USA) at 2.4 GHz with 16 GB of RAM was used.

**Figure 5:**
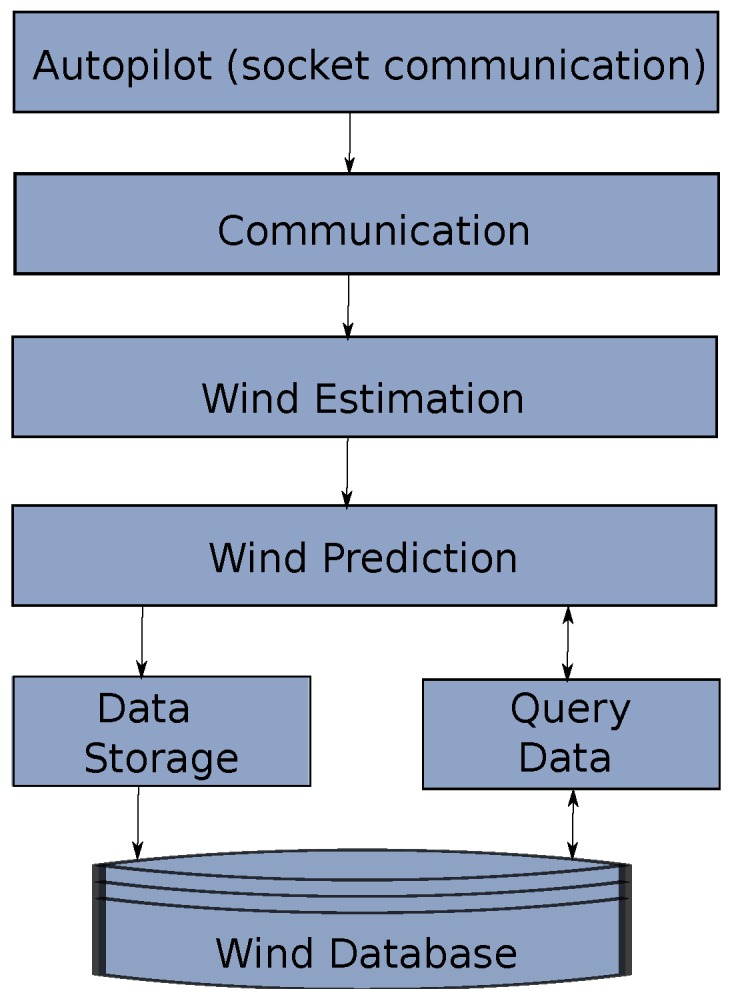
High level software architecture that shows the flow of information of the wind identification system.

The software design has evolved deeply since its conception. Initially the system was created in a multi-platform way with different computing languages interacting at a very high level. The proposed architecture intends to minimize these interfaces at component-level in order to enhance maintainability and upgradeability of the system. In the architecture shown in Figure 5, the autopilot sends information from a request made by the communication module, this information is sent to the wind estimation algorithm that generates wind estimates that will go to the prediction block which uses information from the wind database and also calls the storage module once a prediction is performed.

As it was mentioned before, the designed architecture considered a diversity of programming languages and even various operating systems. The modules communication of this system was done in Linux with pymavlink (MAVLINK (Micro Air Vehicle Communication Protocol) is a communication library for UAS that can pack C-structures over a serial channel and send this packets with other modules. It was originally released in 2009 by Lorenz Meier with a GNU Lesser General Public Licence (LPGL). Pymavlink is a Python implementation of MAVLINK [26]). The wind estimations and the simulation test-bed with the models shown in Section 2 were done using MATLAB, Simulink^®^ (The MathWorks, Inc., Natick, MA, USA). Finally, in order to generate a database, initially the idea was to create comma separated (*.csv) files, however, Structured Query Langate (SQL) was selected to be utilized for Database Accessing and Management, which required Java and C++ connector of SQL.

**Figure 6:**
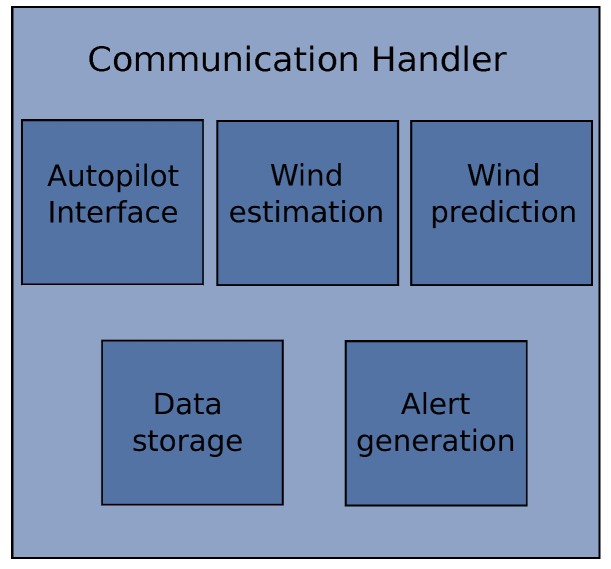
Architecture design with communications handler.

After observing problems in the synchronization of the systems, the solution was to migrate everything to C++ leaving only the database management in JAVA with the MySQL^®^ (Oracle Corporation, Redwood Shores, CA, USA) C++ connector. The concept was to build a modular architecture that runs under a handler that manages the communication between the various modules that interact to identify the wind (see Figure 6).

The modules are the same shown in Figure 5 plus the alerts generation.

An advantage of the modular implementation is that the system can be easily expanded to provide additional functionalities besides the wind identification system. This was thought in order to be able to integrate trajectory optimization functions and controlling modules to follow the desired trajectory.

The details on the implementation of the modules are described in Section 3.1, Section 3.2, Section 3.3 and Section 3.4.

### 3.1. Communication Block and Handler

The explanation of the communications is divided in two parts. The first one, described in Section 3.1.1 analyzes the details of the communication between the three main hardware components: the ODROID, the Pixhawk and the PC with SQL. The second part, Section 3.1.2, explains the details of the communication between the functional software blocks.

#### 3.1.1. Hardware Communication

The hardware communication is performed by a C++ Software implementation derived from the MAVCONN software created by Lorenz Meier as a complementary MAVLINK toolset [27]. The main characteristic is the low latency that allow the communication between processes approximately at 100 microseconds. The system was implemented asynchronously, allowing the data to be sent immediately after it is available. The asynchronous communication is an alternative solution to the widely use polling which is proven to require extra CPU resources because of the context switch. On the other hand, asynchronous design requires minimum CPU resources. Nevertheless, it needs a multi-threaded implementation which is computationally more complex. The ODROID computer allows this type of implementation. Further details of this implementation can be found in [28].

#### 3.1.2. Module Communication

The communication between modules is managed by a handler (see Figure 6). Each module publishes its information at a certain order based on a request and the importance of the information. Therefore, if a module requires priority information the framework will designate this request over others. Table 2 shows the selected requirements in terms of communication rate and an assigned priority based on the importance of its information to other subsystems.

The main advantage of this system is the modularity, since the intention is to have total independence between systems. If there is any communication problem, or the data is proved to be corrupted, this is handled directly by the communication handler which will continue to serve the other functions to preserve the overall integrity.

The processes with highest priority of publishing are the communication request between the ODROID and the PIXHAWK and the wind estimation processes (see Table 2). The first one was based on the publishing rate of the information available from the User Datagram Protocol (UDP) connection with MAVLINK. The second one was based on the computational time that requires the prediction which was subject of previous study in [8,9].

### 3.2. Wind Prediction

The main part of the system consists in a prediction algorithm that is able to recognize wind features (gust and shear) separately. The algorithm performs a statistical analysis to wind velocity estimates in order to determine if a feature is present. First, the module requests a wind estimation to the communication handler. Once it is requested, it stores the data into a temporary database that is going to be used for analysis.

If there are sufficient estimates from the current flight, the system starts a feature detection process by ordering the wind database with respect the UAS altitude. Since the altitude reading vary a lot with time, even in small amounts, the estimates are grouped according to a reference altitude by selecting those altitudes that are close within a given tolerance. Normally the references altitudes are integer numbers and the groups are conformed by those readings between a ±1 m tolerance. At this point the module calls the communication handler in order to request additional measurements. These measurements may introduce significant noise to the system. Therefore, the conditions for the selection of previous measurements include date, time, location, altitude and some weather information. The database query instructions may vary from flight to flight, therefore, the specific conditions and the tolerances value can be specified on a flight-to-flight basis.

For those grouped wind estimates, the module tries to find the corresponding Weibull parameters using GA. If the system finds the Weibull parameters, a most probable wind speed at the corresponding reference altitude is generated. The process is repeated until the local maximum altitude is reached.

At first, the system performs an analysis to determine the presence of a shear, which is a very common feature [23,29]. The wind prediction module tries to find a Prandtl coefficient that minimizes the error between the most probable wind speeds for the reference altitudes. If the estimates are distributed according to the Weibull distribution and there is a Prandtl coefficient *ξ* that produces an acceptable error into the system (during the testing, the Prandtl coefficient was selected when the average error among the different altitudes was ϵ≤ 5 m/s). Then, and alert is triggered and the system recognizes the presence of a shear. Afterwards, the system performs a statistical analysis to determine anomalies (significant jumps) in consecutive wind estimates. These were performed by looking for sudden increases into the running standard deviation of the wind estimates. If there is a sudden increase an initial alert is generated that potentially a discrete gust is identified. If the system is not capable to determine accurately the Weibull parameters of the system, most probable wind speeds cannot be fitted into a shear, and/or the running standard deviation presents drastic changes, i.e., there are continuous increases in the running standard deviation, the system assumes the presence of a continuous gust which triggers a short term Gaussian Regression process in order to characterize the feature. Algorithm 1 describes the insights of the prediction algorithm.

**Algorithm 1** Wind prediction algorithm.    1:  **procedure** RequestWindEstimation    2:  WindEst = CommHandler.Request.CurrentWindVel▹ Request a wind speed estimation (see Equation (2)).    3:  WDb(CommHandler.Request.WVelCount++)= WindEst;▹ Store WindEst to Database.    4:  **end procedure**    5:  **procedure**
DetectFeature(WDb)▹ Requires Wind Database (WDb) with at least 30 elements    6:  Start=False;    7:  **if** WDb.Size ≥30
**then**    8:     Start = True;▹ Start detection of features.    9:  **else**  10:     CommHandler.Alert = InsufficienElements;▹ Wait until DB has sufficient elements.  11:    **end**
**if**  12:    **if** Start==True **then**  13:      WDb = OrderAltitudes(WDb);▹ Order WDb based on altitude.  14:      **for**
i=1←AltMax **do**▹ Check for altitudes 1 m to maximum altitude.  15:        NearAlts = FindNearAltitudes(WDb,*i*,thres);  16:        AdNearAlts = CommHandler.RequestDb(*i*);▹ Additional WDb elements to master Db.  17:        NearAlts = [NearAlts:AdNearAlts];▹ Group elements.  18:        WindVelMP = FindMPWVel(NearAlts)▹ Find most probable wind speed at altitude *i*
m.  19:        MPS(*i*) = Store(WindVelMP);▹ Store the most probable wind speeds.  20:      **end**
**for**  21:      Prandtl = CalcPrandtl(MPS)▹ Calculate Prandtl coefficient from Equation (6).  22:      **if** Prandtl.Exist = True **then**  19:        *ξ* = Prandtl;  24:        CommHandler.Alert = ShearDetected;  25:      **end**
**if**  26:      i++;  27:      **if** Exist(Prandtl) = False **then**  28:        DetectJumps(WDb.Velocity,Std(WDb.Velocity))▹ Look for jumps in running std. dev.  29:      **end**
**if**  30:      **if** CommHandler.Request.Alert.JumpDetected = True **then**  31:        JumpCounter++;  32:      **end**
**if**  33:      **if** JumpCounter≥threshold **then**  34:        Commhandler.Alert = ContGustDetected;▹ Is a continuous gust.  35:      **else**  36:        Commhandler.Alert = DiscGustDetected;▹ Is a discrete gust.  37:      **end**
**if**  38:      **if** CommHandler.Request.Alert.DiscGustDetected = True **then**  39:        Gust = DetectJumps.Jumpsize  40:      **else**
**if** CommHandler.Request.Alert.DiscGustDetected **then**  41:        ContGust = PerformGaussianRegressionWDb▹ See note **.  42:      **end**
**if**  43:    **else**  44:      CommHandler.Alert = NoFeatureDetected;▹ No feature was detected.  45:    **end**
**if**  46:  **end procedure**** The system may perform a long-term and a short term prediction. For this research activity only the short-term which is a Standard GP regression. The non-homogeneous GP regression requires a vast amount of information which is part of future activities.

The algorithm that is used to group the altitudes based on a reference is shown in Algorithm 2.

**Algorithm 2** Grouping near altitudes algorithm.  1:  **procedure** FindNearAltitudes(WDb,alt,thres)▹ Find altitudes in WDb close to alt.  2:    Counter = 0;
  3:    **for**
i=1←WDb.Size **do**
  4:       **if** alt-thres≤WDb(*i*).Altitude≤alt+thres **then**  5:          NearAlts(Counter++) = WDb(*i*);▹ Store whole WDb.  6:       **end**
**if**  7:    **end**
**for**  8:    **return** NearAlts;  9:  **end procedure**

The determination of the most probable wind speed at a given altitude is described in Algorithm 3.

**Algorithm 3** Weibull parameter calculation algorithm.    1:  **procedure** FindMPWVel(NearAlts)▹ Find altitudes.    2:    κ= CalcKappa(NearAlts)▹ Calculate shaping parameter using GA.    3:    $\nu =\frac{1}{\mathrm{Mean}(\mathrm{NearAlts})}\Gamma \left(1+\frac{1}{\kappa }\right)$▹ Calculate scaling parameter from Equation (17).    4:  **end procedure**    5:  **procedure** CalcKappa(Altitudes)▹ GA Implementation (see note***).    6:    PopulationSize = 50;    7:    FunctionTolerance = $1\times 10^{-3}$;    8:    MaxGenerations = 100;    9:    CrossOverFraction = 0.8;  10:    StdAlt = Std(NearAlts);▹ Calculate standard deviation.  11:    MeanAlt = Mean(NearAlts);▹ Calculate mean.  12:    PopKappa == rand(PopulationSize);▹ Initialize with random population.  13:    **while**
ϵ> FunctionTolerance **do**  14:       **for**
j=1←PopKappa.Size **do**  15:          Results(*j*) = ObjFunc(PopKappa(*j*),StdAlt,MeanAlt);▹ Evaluate Objetive Function.  16:       **end**
**for**
  17:       Parents = Selection(Results,PopKappa);▹ Selection of elements for newGeneration Equation (16).  18:       Reproduction(Parents,PopKappa,MaxGenerations;)▹ Creation of new population.  19:       Crossover(CrossOverFraction);▹ Scattered crossover function.  20:       Migration();▹ Gaussian Mutation function.  21:    **end**
**while**  22:  **end procedure***** The selected parameters were the same ones utilized in previous implementations [8,9].

Algorithm 4 describes the calculation of the Prandtl coefficient once the system detects a stable running standard deviation.

**Algorithm 4** Wind Prediction Algorithm.    1:  **procedure** CalcPrandlt(WindSpeeds)▹ Determine Prandtl Coefficient.    2:   Prandtl.Exist = False;▹ Initialize values.    3:   Prandtl.Value = 0;    4:   **for**
m=0.01←1
**do**▹ Evaluate potential Prandtl coefficient.    5:      **for**
l=1←MaxAlt **do**▹ Evaluate for altitudes in MPWS.    6:         CalError = ComparePrandtlValues    7:      **end**
**for**    8:      **if** Mean(Error)≤thres and Std(erorr)≤thres **then**    9:         Prandtl.Exist = True;▹ If coefficient gives a minimum average error.  10:         Prandtl.Value = m;▹ Prandtl coefficient is *m*.  11:         break;  12:      **end**
**if**    4:   **end**
**for**    4:   **return** Prandtl  15:  **end procedure**

Algorithm 5 is used for detection of anomalies in the running standard deviation.

**Algorithm 5** Jump detection algorithm.  1:  **procedure** DetectJumps(WindSpeeds)▹ Look for jumps in running std dev.  2:   PrevStd = Std(WindSpeeds(k − 1));▹ Look for previous std. dev.  3:   DiffStd = PrevStd-Std(WindSpeeds)▹ Difference between std. deviations.  4:   AcumDiffStd(count + 1) = DisffStd  5:   **if** DiffStd ≥ thres **then**▹ If error is bigger than threshold.  6:      CommHandler.Alert = JumpDetected  7:      JumpSize = Mean(AcumDiffStd)▹ Estimate the size of the jump.  8:   **end**
**if**  9:  **end procedure**

### 3.3. Data Storage and Wind Database

An important part of the designed system is the storage and management of the information. This information is the one generated by the estimation and the prediction modules, and also the one generated by the autopilot (vehicle state: position, velocity, acceleration).

SQL is selected as a means of the generation, storage and management of the database. This was because SQL is a standardized language for database management. SQL is a language by itself, therefore, it requires an interface with the wind identification system. The SQL system that is selected for this research is MySQL^®^ and the interfacing between the database and the wind identification comm-handler is done trough the MySQL^®^ C++ connector. This allows the generation of C++ commands that will read and write information from any SQL database.

**Figure 7:**
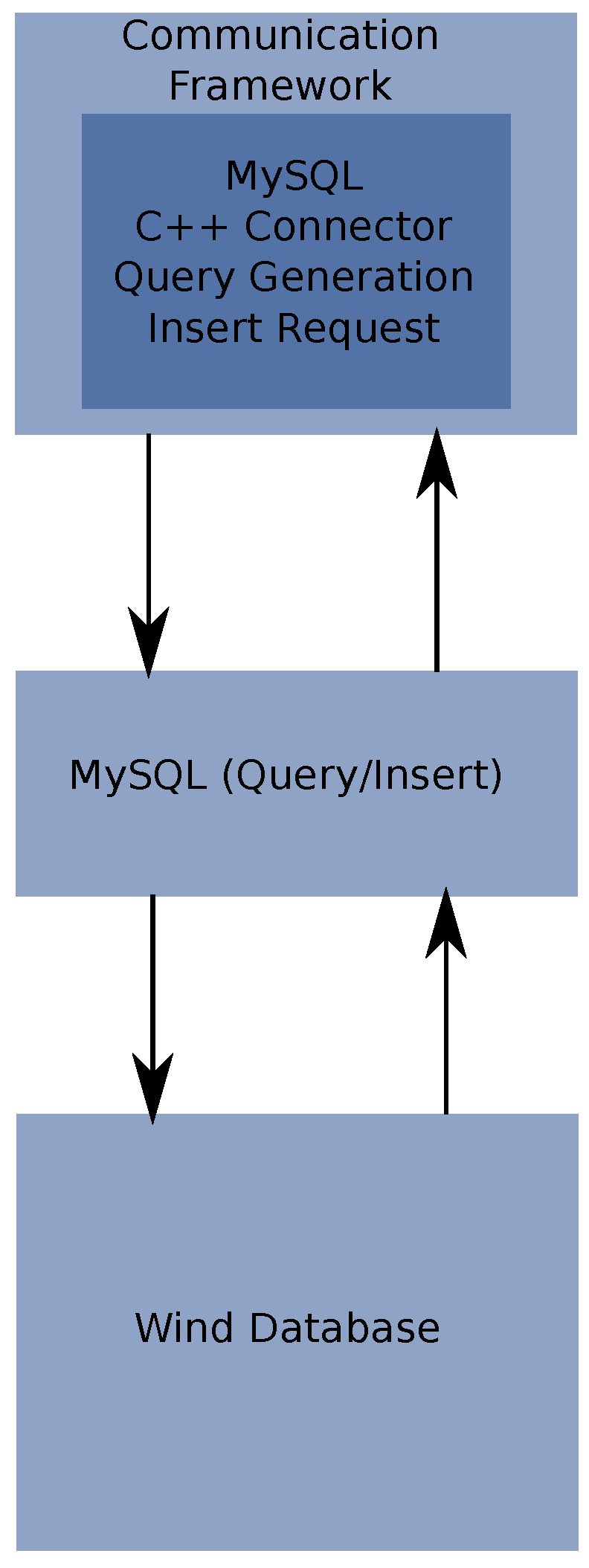
Database interfacing with communication handler.

The communication scheme is shown in Figure 7.

Figure 7 illustrates the two modules that are required to interact with wind database. One is the MySQL C++ connector and the other the MySQL system, which have to be compatible with the used operating system.

The algorithm for accessing the database, perform a query of the useful data and write the generated data for the modules consists in a series of calls to the SQL connector which needs to open a Connection to the SQL server and then to execute an update or a query to the database based on the parameters that are needed. This is described in Algorithm 6.

**Algorithm 6** Database Access, Query and Writing Algorithm.    1:  **procedure** WaitForRequest(DBReq)    2:  **if**
DBReq = Write **then**    3:     WriteDB(DB);    4:  **else**
**if**
DBReq = Query **then**    5:     Query(DB,Cond);    6:  **else**    7:     TriggerException;    8:  **end**
**if**    9:  **end procedure**  10:  **procedure**
WriteDB(DB,WindVector)  11:  Con→ CreateDriver();▹ Create Database Driver  12:  Con→GetDriverInstance();▹ Used to get the Driver Instance and Load the DB.  13:  Con→setSchema(DB)▹ Set the DB to write to  14:  Stmt→WindVector  15:  **end procedure**  16:  **procedure**
QueryDB(DB,Cond) State Con→ CreateDriver();  17:  Con→GetDriverInstance();  18:  Con→setSchema(DB);  19:  execute;  20:  stmt→executeQuery(Condition);▹ See note below.  21:  **end procedure**

In the previous algorithm, the query is based on the location the time of the year. Since the location of a typical mission may not vary the data should be valid, however a future step is to include Meteorological Terminal Aviation Routine Weather Report (METAR) weather reports to the query so that it only looks for wind predictions and estimations performed in similar meteorological conditions.

### 3.4. Alert Generation Module

A complementary part of the estimation module is the alert generation algorithm. This part illustrates what kind of alerts need to be triggered internally to the system and to the user so that it can take a decision. These alerts are related to the detection and the uncertainty of a feature . In addition, there are different alerts that are generated inside each module related to the information that each module produces, including the generation of software exceptions.

Table 3 shows the main alerts that are generated once a feature is detected, the variable type of the alert and the priority.

The alert priority value aids on determining how often an alert is generated. The goal of the system is to alert to other modules the presence of a feature and to display these alerts in the ground station.

The prediction time window (*τ*) requires additional computational resources. If a discrete gust of a shear is detected, and the running standard deviation remains stable, a prediction time window alert is not required (for computation purposes is considered as infinite). However, if a continuous gust is detected the time window of the prediction goes critical depending to the behavior of the difference between the prediction and the estimation. If the running standard deviation of this difference is bounded, the prediction window can be slightly increased. If there is an unbounded behavior, then the system stays at its initial value ($\tau_{0}=$ 5 s), based on the results published by Passner et al. [30].

## 4. Simulation and Experimental Results

This section presents the preliminary validation results of the system obtained with simulations and Autopilot/Framework SITL experiments with real telemetry data obtained in four flights which took place in the Seville Metropolitan Area in Andalusia (Spain).

### 4.1. Simulation Test Bed Description

MATLAB^®^ and Simulink^®^ has been utilized for simulation. The Aerospace toolbox contains wind-model blocks of shear, discrete and continuous gusts. In addition the AeroSim^®^ blockset has been utilized to generate 6DOF model of a small UAS.

**Figure 8:**
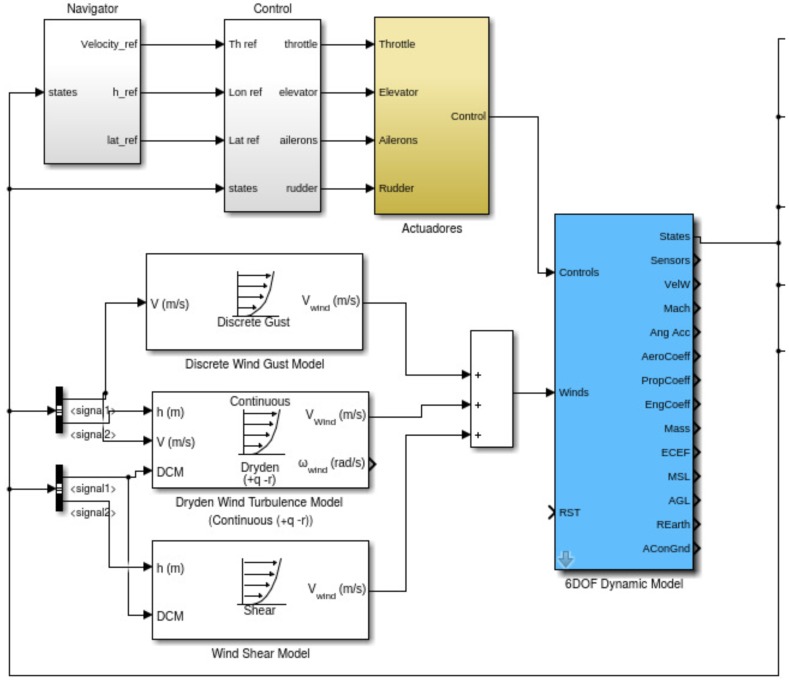
Simulink model of the simulation environment for the wind identification system.

The 6DOF model utilized together with the wind dynamic model blocks are shown in Figure 8.

The blue block shows the 6 DOF dynamic model and the white blocks show the wind dynamic model. In addition, there is an actuator block that corresponds the dynamic model of the actuators. There are two additional blocks that show the navigation and the control modules which are a series of nested Proportional-Integral-Derivative (PID) controllers.

Table 4 show the characteristics of the computer used in order to perform the simulations.

The corresponding trimming parameters for a typical flight condition [31] are utilized in the simulation are shown in Table 5.

**Figure 9:**
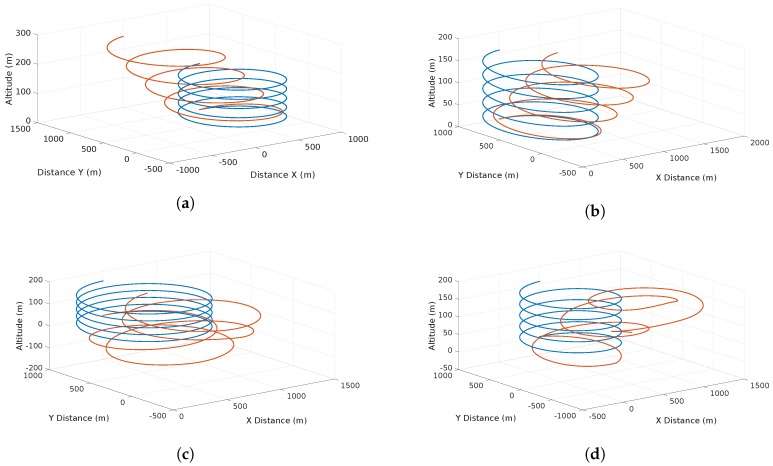
Effects of different simulated features on the vehicle trajectory: (**a**) the effect of a shear wind with increasing deviation as altitude rises; (**b**) the effects of a discrete gust with a constant deviation on the trajectory in a single direction; (**c**) a chaotic deviation due to the effects of a continuous gust; and finally (**d**) the total effects of the wind present at the same time.

The scenario considers a planned helix flight ascending trajectory. Once the vehicle starts its flight, the trajectory is under the influence of different wind types. Two scenarios are considered. The first one considers each feature separately (shear, discrete gust, continuous gust) and the second one considers all features at the same time. The purpose of this simulation is to prove the ability of the system in controlled conditions of detecting the features separately. Figure 9 depicts the wind effects on the trajectory.

### 4.2. Simulation Results

**Figure 10:**
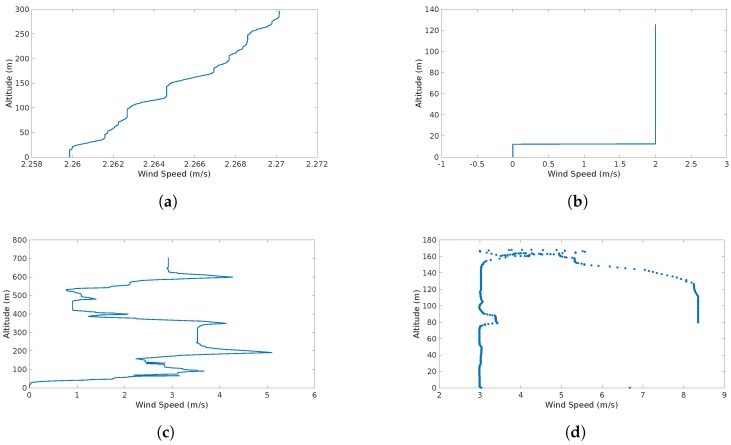
Wind Speed/Altitude maps of the different simulation scenarios: (**a**) the wind shear as an increase of wind speed with altitude; (**b**) a sudden increase in wind speed at a certain altitude (discrete gust); (**c**) a continuous gust with a chaotic effect and rapid increases and decreases of wind speed; and (**d**) the sum of the three effects.

The detection capabilities of the system are illustrated Figure 10. It shows the information that feeds up the system and how it will detect and identify the different features.

Figure 10d shows two trends (vertical successions of wind velocity points). One in which the wind speeds are distributed uniformly across altitudes with a mean value of approximately 3 m/s. At 100 m one can observe another succession of points with a mean value of approximately 8.2 m/s. This produces a sudden increase (jump of aproximately 5 m/s) in the standard deviation which triggers an alert of gust detected and forces the system to characterize two separate distributions, one after and one before the gust.

**Figure 11:**
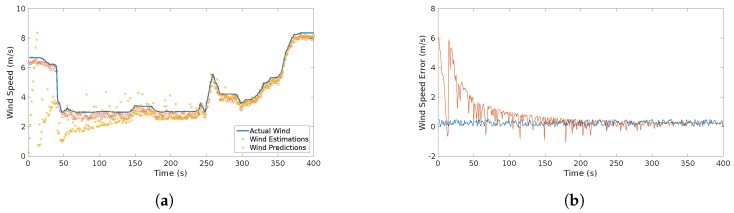
Actual, estimated an predicted wind speed (**a**) and wind speed error (**b**) in the considered scenario. The predicted wind starts with high dispersion, however, it converges to the actual value within 100 s.

The results of the wind estimates and predictions of the wind are show in Figure 11.

In this flight, an alert of a continuous gust detected was triggered almost immediately (at approximately 20 s). In addition, there was an alarm of two detected gusts: one occurred at approximately 40 s and the other occurred at 250 s. This coincides with the jumps, abrupt changes of the wind speed, that can be seen in Figure 11.

### 4.3. Software-in-the-Loop Experiments

The wind identification system has been functionally tested with real telemetry data. The data were fed into the system using Mavlink interfacing with a Ground Control Station as in [32]. The sensor information was transmitted to the wind identification system at a rate of 0.5 Hz. Nevertheless the communication framework demands varied in frequency due to the asynchronous scheme. The transmission to the system does not match the actual duration of the telemetry log, it was truncated once the platform had landed.

**Figure 12:**
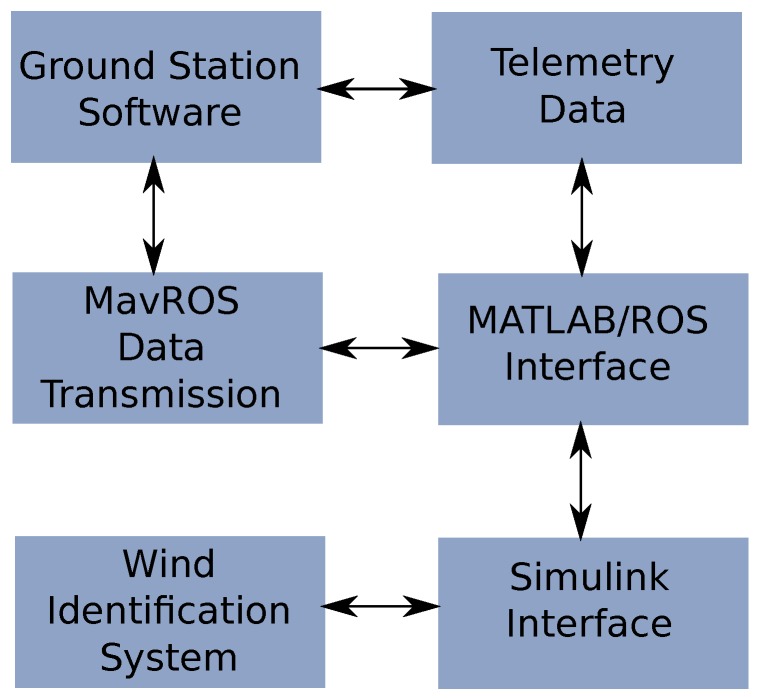
Information flow for on-the-loop experiments of the wind identification system. The blocks show the multi-platform interfaces that allowed the validation tests.

The test-bed architecture uses a MATLAB^®^- Mavlink interface implemented in the Robotic Operating System (ROS). The wind identification system interfaces with MATLAB^®^ through a series of S-Functions. This concept is illustrated in Figure 12.

**Figure 13:**
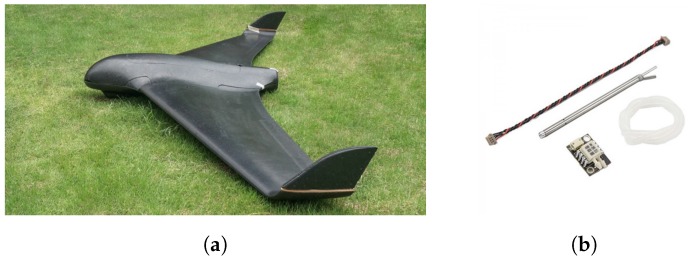
Sensor and UAS platform utilized in experiments. (**a**) UAS SkyWalker X8, (SkyWalker Technology Co., Ltd, Wuhan, China) with carbon fiber frame equipped with a 12 × 6 prop with 2 × 20 g servomotor; (**b**) Digital airspeed sensor utilized in experiments which contains a 4525DO sensor (TE Connectivity Ltd., Schaffhausen, Switzerland) which enables a resolution of 0.84 Pa [33].

The platform and the airspeed sensor in which the experiments were performed is shown in Figure 13a.

Table 6 presents the characteristics of the platform shown in Figure 13a.

The vehicle was equipped with an APM2.6 autopilot (3D Robotics, Berkeley, CA, USA) with the airspeed sensor illustrated in Figure 13b.

### 4.4. Software-in-the-Loop Experiments Results

The information of the flights performed in the Seville Metropolitan Area (Brenes) is shown in Table 7.

**Figure 14:**
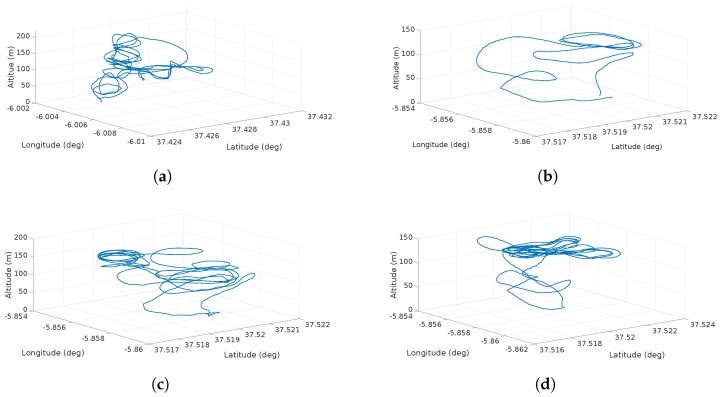
UAS Trajectory for validation of the wind information system. (**a**) shows a medium altitude with few spirals; (**b**) shows the shortest flight; (**c**) shows a flight with spirals performed at an altitude of 120 m; and (**d**) shows a flight with wide spirals at an altitude of 120 m.

Figure 14 depicts the flight trajectories of the scenarios described in Table 7. The first flight shows 8 maneuvers performed at different altitudes. The other three flights consisted on takeoff, several spirals at a target altitude and then the descent and landing.

**Figure 15:**
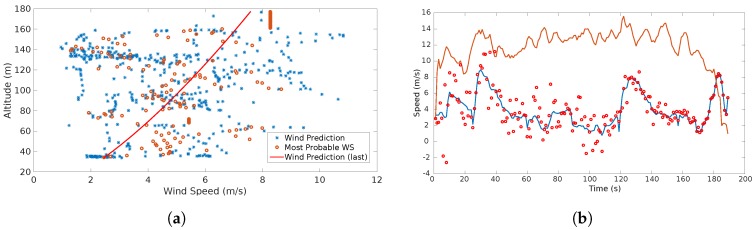
(**a**) shows the estimation, most probable wind speeds and last shear wind prediction generated throughout the flight; (**b**) indicates the wind speed estimation (blue line), wind speed prediction (red dots) and airspeed (orange line).

Figure 15 depicts the results obtained from Experiment 1:

The red dots in Figure 15 indicate the predicted wind speed. The blue line represents the estimations which were obtained with the direct computation method presented in [2,8,9]. A continuous gust alarm was generated almost at the beginning of the flight due to the continuous changes in wind speed over time.

**Figure 16:**
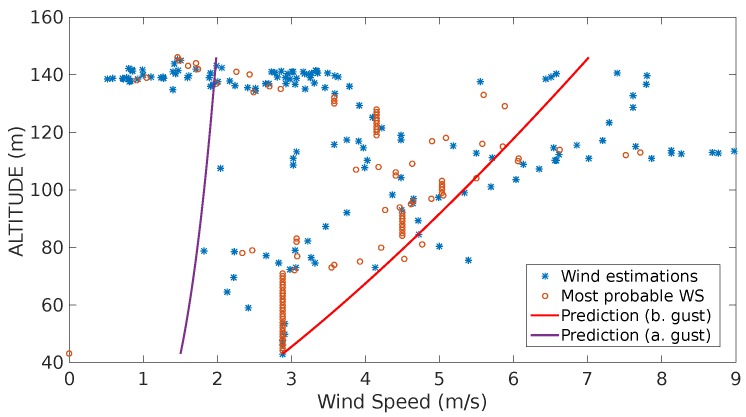
Estimation, most probable wind speeds and last wind prediction generated throughout the flight shown in Figure 14b.

The second experiment (see Figure 16) shows a significant decrease of the estimates estimation as the UAS reaches its maximum altitude. The system interpreted this tendency as a negative gust, i.e., a sudden reduction of the wind speed. Once the system generates the corresponding alarm and the running standard deviation of the estimates stops growing, the system starts characterizing a second shear which is represented by the purple line.

**Figure 17:**
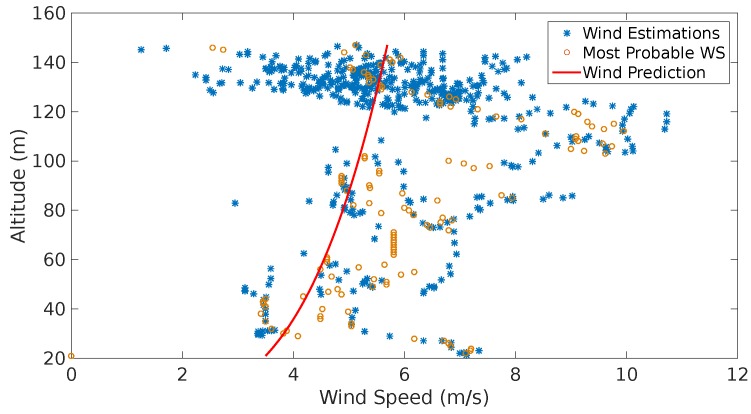
Estimation, most probable wind speeds and last wind prediction generated throughout the flight illustrated in Figure 14c.

**Figure 18:**
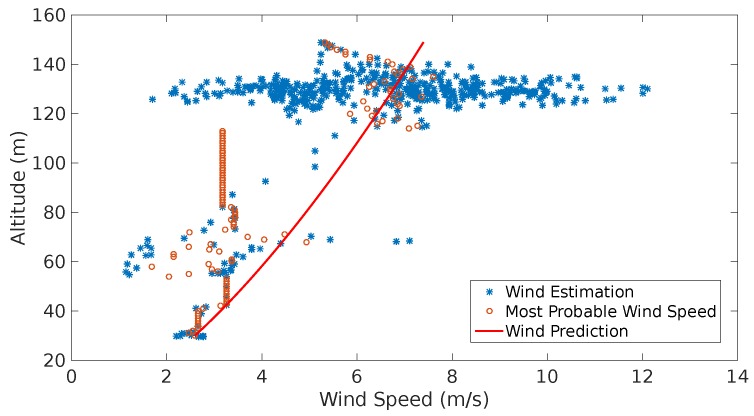
Estimation, most probable wind speeds and last wind prediction generated throughout the flight depicted in Figure 14c.

Experiments 3 and 4 (see Figure 17 and Figure 18) show a very similar behavior. The wind speed estimates show higher values as the altitude grows. The high concentration of estimations of the wind speed between 120 m and 140 m altitude show big dispersion which suggests that the UAS maneuvers affect the speed reading as the accelerometers and the GNSS speed readings affect the computation of the estimates. More testing is required to support this hypothesis.

The average Weibull shaping parameter, *κ*, Weibull scaling parameter, *ν*, and the calculated Prandtl coefficient, *ξ*, are shown in Figure 14d.

Note that in scenario 2 of Table 8 two values of shaping parameter, scaling parameter and Prandtl coefficient appear for scenario 2. They correspond to two different shear characteristics detected before and after the presence of a discrete gust.

**Figure 19:**
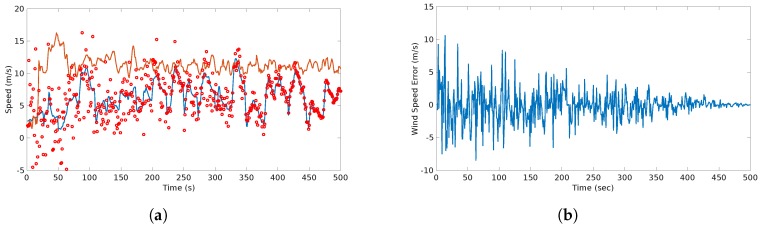
Predicted (red circles) vs. Estimated wind speed (blue line): (**a**) shows the difference between the estimation and the prediction of the wind speeds with the airspeed (orange) as a reference; (**b**) shows the actual difference between these two quantities which appears to be bounded and shows a normal behavior.

Figure 19 shows the difference comparison between the estimations and the predictions of the wind speed magnitude. It is important to consider that it is the difference, therefore, it cannot be considered as an absolute error. However it aims to prove that this difference is bounded since it considers local measurements.

The difference analysis between all the flights together showing the mean $\mu (W_{e})$ and the standard deviation $\sigma (W_{e})$ are shown in Table 9.

## 5. Results Discussion

In the simulation results, the effects of the wind features (continuous and discrete gusts and shear) are shown in Figure 9. It is observed that single or multiple features can affect the trajectory without any sort of drifting compensation. Figure 10 illustrates the effects of the features in wind speed/altitude charts. The system is able to identify this feature from the summed effects plot shown in Figure 10d. However, at this stage noise is not considered and it can have a significant impact to the plot, so the estimation process has to be accurate enough since it is the only input to the wind identification system. The case shown in Figure 10d is analyzed in detail since the effects of each feature separately were analyzed in [8,9].

The results plotted in Figure 11 show the behavior of the estimation and prediction processes. The estimation process considered a slight Gaussian noise which is typical for airspeed sensors [2]. The error of the estimation process is bounded as observed in Figure 11b which is indeed the expected behavior and is consistent with the result presented in [2,8,9]. On the other hand, the prediction shows a different behavior. At the beginning and up to 70 s there is a considerable dispersion of the prediction due to the assumption that only a shear feature is present since the beginning. Then, the system starts identifying other features. At 40 s a rapid change is observed which triggers a discrete gust alarm. Up to that point the system starts converging and the predictions with a variable window start happening. Since there is a continuous gust during the entire scenario, the system utilizes Gaussian regression to start predicting the behavior of the plot. Even though there are rapid changes at some parts, the identification of the discrete gust minimizes the effect in the Gaussian regression. The prediction error shows a gradual decrease up to the point that it follows a similar behavior than the estimation. This concludes that the prediction error tends to be bounded as more data is fed to the system.

The SITL testing illustrates four scenarios with actual airspeed measurements. Figure 14 shows different paths with different maneuvers at various altitudes. These scenarios are very helpful to comprehend how the noise of the airspeed measurements affects the estimation. However, these results have to be treated carefully since there is no ground-truth and intend to validate the functionality of the system only. Full validation of the results needs to come from full validation and verification with Software, and Hardware-In-The-Loop and extensive flight testing in different conditions. The intention of the upcoming testing activities is to prove every aspect of the system and to analyze how the obtained results support the hypotheses on the wind identification problem. Nevertheless, current preliminary results prove that the wind estimates behave statistically as expected, in cases of shear and discrete gusts, estimations and predictions are Weibull distributed and they keep following the Prandtl law with a low increasing running standard deviation. In the case of continuous gusts, the short-term regression has proved to be accurate, keeping the running standard deviation of the predicted wind bounded throughout the flight.

In the scenario shown in Figure 14a the results of the prediction and estimation process show a very dispersed behavior (see Figure 15a). The system triggers a continuous gust alarm and the prediction process employs a GP Regression. Note that a shear is identified (red line), however, this is not taken into account in the prediction, since the system always assumes that once there are sufficient wind estimates there is a shear. Once the continuous gust is detected and during the computation of the GP regression, the system stops calculating the shear characteristics releasing computational load as no GA is performed.

The scenario shown in Figure 14b shows two shear features that are identified due to a sudden change of wind speed that occurs at 40 m. Since a discrete gust was detected the system tries to identify this two features. It is observed that there is are three points that the most probable wind speeds looks constant. This is due to the lack of measurements possibly by a communication error between the system and the ground station.

The remaining two flights (see Figure 14c,d) show a very similar behavior at high altitude. Nevertheless in the fourth flight there is a lack of airspeed measurements and the most probable wind speed is assumed to be constant. However the actual prediction (red line) shows what in reality the airspeed has to behave. Since a lot of spiral maneuvers were performed, the system has a vast amount of estimations over an altitude of 120 m, however, there is a substantial dispersion that follows the Weibull distribution allowing the generation of coherent most probable wind speeds and to treat this measurements as part of a wind shear feature.

The prediction models illustrated in Figure 16 show two characterized shear predictions as a results of the identification of a gust. The system performs this interpretation as the estimates from 40 m to 120 m show a tendency of a sustained growth, however when the vehicle passes 130 m most of the estimates go back to values around 2 m/s.

In Figure 17 one can observe dispersion in the wind estimates from the lowest altitude up to 120 m even with a few measurements at some altitudes, e.g., between 50 m and 80 m. However, the system was able to identify a Prandtl coefficient to characterize an average shear. The last prediction (red line) is moved ot the left as most of the available estimates were above 120 m. In higher altitudes, the measurements are dispersed (2 m/s to 8 m/s), however, the alerts generated indicate that the system was able to find Weibull parameters for these measurements. This indicates that the system might require an adjustment of the tolerances for continuous gust detection.

Figure 18 depicts a more stable behavior across different altitudes. Most of the measurements are concentrated above 120 m, however, with the measurements below those altitudes the system is able to produce a solution in which a wind shear is identified. The measurements above 120 m show big dispersion, possibly due to sensor noise, however these were proved to be Weibull-distributed, hence, the system was able to produce a set of most probable wind speeds and a prediction tendency.

For the last scenario, a comparison between estimation and the prediction can be observed in Figure 19. The error between the predictions and estimation is bounded since the very beginning, mainly to the absence of continuous gust features. This results can be confirmed with the study of the dispersion of this difference that is shown in Table 9.

## 6. Conclusions and Future Work

This paper presents the integration of a wind identification system using small UAS. It describes the high and low level architecture and provides a initial validation with simulations and software-in-the-loop testing.

The system architecture integrates different components at various levels, and presents significant advances from the previous research activities presented in [8,9]. In terms of hardware, the proposed system uses COTS components which help on cost efficiency without the sacrifice of functionality or reliability mainly due to the system characteristics and current state-of-the-art. On the other hand, the software has a decentralized integration at system and component level due to the development of a communication handler. This manages the information exchange between the different modules of the system. The main advantages of this communication scheme are the separation between different functional modules, which ensures the upgradeability and the module dependencies. Also the possibility can be easily expanded by adding other functional modules, such as trajectory generation/optimization. Another factor considered in the design was the possibility of asynchronous communication between blocks. This is an important requirement due to the possible variation on the processing time for different modules regardless if the variation is generated at a software or hardware level.

The core function of the system which is the prediction module is described and presents significant improvements from the previous research activities. The algorithm has unique way to characterize the features as it intends to find statistical key values that will lead to the identification of a feature. The system now triggers alarms to the communication handler and the sub-procedures were clearly defined and tested. Other modules such as the communication handler and the database management and query were also analyzed. The implemented algorithms work asynchronously and even though the computational demand may be significant to the use of nested loops and complex algorithms such as GA, they do not impact the prediction computation since the algorithms intend to find a minimum of variables. The most costly algorithm is the database management as it intends to do a smart search of accumulated data from previous flights. This is not an issue since it is done in a separate dedicated computer. Even though there is no information from the wind database, the system is able to produce results as it depends only in the current estimates.

In terms of the wind speed estimation and wind speed prediction validation, the system was tested with both simulations and software-in-the-loop. In the simulations, results indicate that the wind identification system is capable of identifying the different features and eventually converges to the actual wind field within variable time windows. However, the accuracy varies according to the identification of other features. Therefore, as clearer features are identified, the convergence time is reduced together with the error magnitude and dispersion. In the SITL testing, the system exhibits dispersion on the wind estimates which is mainly attributable to the noise from the airspeed sensor. The system estimates were Weibull-distributed in altitudes on which the aircraft remains for longer periods and presented inaccurate predictions at altitudes in which the aircraft has low or null density of measurements. The presented results lead to the conclusion that the system fulfills the design requirements and provides the identification of separated wind features which could be really useful for trajectory planning and optimization. The novelty of the system relies in two main aspects: first the architecture with a upgradeable system with minimum module dependency and secondly the information that the system generates since the identification of separated wind field features could easily be used for efficient trajectory planning, for instance in dynamic soaring.

Future work includes details on the generation of 3D wind maps and a complete validation and verification of the system at system and component levels, as well as on-board/real-time testing of the system. This paper intends to present a detailed description and the initial stages of validation and verification of the system. The full testing, including hardware-in-the-loop and on-board testing activities and the integration of the mapping feature will be subject of a further publication (Part 2) which will provide results at system and component level in terms of accuracy and reliability and a detail analysis of the computational cost of the different methods. In addition, upcoming work includes the integration of a trajectory generation module and the generation of control commands to follow the wind-efficient trajectories which ultimately is derived from the objective of increasing substantially the flight duration in a given mission. 

## Figures and Tables

**Table 1:** ODROID-C2 Specifications from [25].

CPU	Amlogic S905 SoC, 4×ARM Cortex-A53 2 GHz, 64 bit ARMv8 Architecture @28 nm
RAM	2 GB 32 bit DDR3 912 MHz
Flash Storage	Micro-SD UHS-1 @83MHz/SDR50, eMMC5.0 storage option
ADC	10 bit SAR 2 channels
Size	85 mm × 56 mm (3.35 inch × 2.2 inch)
Weight	40 g (1.41 oz)

**Table 2:** Communication Scheme of Handler. The publishing rate which was determined based on the results found in [9] and the priority based on the system requirements.

Function	Publishing Rate	Priority
APM Comm Request	2 Hz	1
Wind Estimation	1 Hz	1
Wind Prediction	0.25 Hz *	2
Database Management	0.2 Hz	3
Database Search	0.25 Hz	2
Alert Generation	0.1 Hz	4

* This rate was selected due to the current time required to scan the wind database. Future work will optimize this rate allowing the generation of predictions at a lower rate.

**Table 3:** Alerts, data types and the priority values.

Alert Type	Data Type	Priority Value
Feature Is Present	Boolean	1
Wind Shear Detected	Boolean	1
Discrete Gust Detected	Boolean	2
Continous Gust Detected	Boolean	3
Prediction Time Window	Integer	2
Uncertainty Level	Double	4

**Table 4:** Simulation computer relevant characteristics.

Component	Specification
CPU	Intel Core i7-5500U CPU 2.40 GHz × 4
RAM	15.6 GiB
Graphics	Intel HD Graphics 5500 (Broadwell GT2)
OS Type	64-Bit
OS	Ubuntu 16.04 lts

**Table 5:** Selected Trimming Parameters.

Parameter	Value
Trim airspeed	25 m/s
Trim altitude	150 m
Trim bank angle	$0^{o}$
Fuel mass	2 kg
Flap setting	0

**Table 6:** Skywalker Characteristics.

Parameter	Value
Wing Span	2122 mm
Wing Area	80 $d^{2}m$
Max Payload	2 kg
Center of Gravity	435 mm away from nose

**Table 7:** Experiments information.

	Flight 1	Flight 2	Flight 3	Flight 4
Duration	521 s	315 s	631 s	749 s
Distance Traveled	5.1 km	3.7 km	6.3 km	7.4 km
Maximum Altitude	179 m	125 m	134 m	146 m

**Table 8:** Main SITL outputs (single run).

Scenario	μ (κ)	μ (ν)	μ (ξ)
1	4.24	1.9825	0.6628
2	1.1579 & 3.1425	0.4531 & 1.6671	0.5314 & 0.6628
3	2.9820	0.8349	0.3124
4	5.7425	0.9623	0.7614

**Table 9:** Mean and standard deviation of difference between the wind speed estimations and the wind speed predictions.

Flight	$\mu \;(W_{e})$	$\sigma \;(W_{e})$
1	1.985 m/s	2.54 m/s
2	1.197 m/s	1.21 m/s
3	0.932 m/s	0.74 m/s
4	0.854 m/s	0.27 m/s

## Abbreviations

The following abbreviations are used in this manuscript:

ADC	Analog to Digital converter
AOA	Angle of Attack
APM	Autopilot Module
COTS	Commercial-Off-The-Shelf
CPU	Central Processing Unit
DB	Database
EKF	Extended Kalman Filter
GA	Genetic Algorithm
GEV	Generalized Extreme Value
GNSS	Global Navigation Satellite System
GP	Gaussian Proccess
IMU	Inertial Measurement Unit
LPGL	GNU Lesser General Public License
PID	Proportional, Integral, Derivative
RAM	Random Access Memory
SITL	Software-In-The-Loop
SQL	Structured Query Language
TN	Truncated Normal (Distribution)
UAV	Unmanned Aerial Vehicle
UDP	User Datagram Protocol
UKF	Unscented Kalman Filter
